# Comment on Foti et al. Identification of Achille’s Tendon Tears: Diagnostic Accuracy of Dual-Energy CT with Respect to MRI. *J. Clin. Med.* 2024, *13*, 4426

**DOI:** 10.3390/jcm13237320

**Published:** 2024-12-02

**Authors:** Nicola Maffulli, Filippo Spiezia

**Affiliations:** 1 Centre for Sports and Exercise Medicine, Queen Mary University of London, London E1 4DG, UK; n.maffulli@qmul.ac.uk; 2 Department of Health Science, UNIBAS, Via dell’Ateneo Lucano10, 85100 Potenza, Italy; 3 Department of Orthopaedic and Trauma Surgery, Ospedale San Carlo, Via Potito Petrone, 85100 Potenza, Italy

We read with great attention, and fully enjoyed, the work by Foti G [1]. It is always interesting to read about novel modalities in the diagnostic assessment of pathologies which have classically been considered to be difficult to assess. We are, however, somewhat puzzled at the fact that such studies are being performed and seriously concerned that simple, valid, reliable methods are not being considered.

DECT involves ionizing radiation [2]. Various applications of dual-energy CT (DECT) have recently been investigated, but only limited data are available regarding the radiation dose associated with DECT imaging [3], and this technology is not widely available [4]. Also, this imaging tool is characterized by technical complexity in terms of the different ways to acquire images and the several algorithms that can be applied in daily clinical practice or research [2].

We are saddened that the authors seem to have ignored that clinical examination provides all the information a clinician needs to formulate a diagnosis of Achilles tendon rupture [5,6,7,8], as shown more than a quarter of a century ago and confirmed by other studies [9,10,11].

Several clinical tests have been described, and we have reported in an unequivocal fashion that when two clinical tests indicate a tear, then the diagnosis is certain, using MRI, diagnostic ultrasound, and surgical appearance as the gold standards [12,13,14]. 

In conclusion, while we welcome studies which detail novel diagnostic modalities, if such modalities are invasive, costly, and not widely available and do not add to clinical diagnosis, perhaps we should be more cautious in recommending them.
